# Energy Load Forecasting Using a Dual-Stage Attention-Based Recurrent Neural Network

**DOI:** 10.3390/s21217115

**Published:** 2021-10-27

**Authors:** Alper Ozcan, Cagatay Catal, Ahmet Kasif

**Affiliations:** 1 Department of Computer Engineering, Akdeniz University, Antalya 07070, Turkey; alperozcan@akdeniz.edu.tr; 2 Department of Computer Science and Engineering, Qatar University, Doha 2713, Qatar; 3 Department of Computer Engineering, Bursa Technical University, Bursa 16330, Turkey; ahmet.kasif@btu.edu.tr

**Keywords:** dual-stage attention-based recurrent neural network, time series forecasting, energy consumption prediction

## Abstract

Providing a stable, low-price, and safe supply of energy to end-users is a challenging task. The energy service providers are affected by several events such as weather, volatility, and special events. As such, the prediction of these events and having a time window for taking preventive measures are crucial for service providers. Electrical load forecasting can be modeled as a time series prediction problem. One solution is to capture spatial correlations, spatial-temporal relations, and time-dependency of such temporal networks in the time series. Previously, different machine learning methods have been used for time series prediction tasks; however, there is still a need for new research to improve the performance of short-term load forecasting models. In this article, we propose a novel deep learning model to predict electric load consumption using Dual-Stage Attention-Based Recurrent Neural Networks in which the attention mechanism is used in both encoder and decoder stages. The encoder attention layer identifies important features from the input vector, whereas the decoder attention layer is used to overcome the limitations of using a fixed context vector and provides a much longer memory capacity. The proposed model improves the performance for short-term load forecasting (STLF) in terms of the Mean Absolute Error (MAE) and Root Mean Squared Errors (RMSE) scores. To evaluate the predictive performance of the proposed model, the UCI household electric power consumption (HEPC) dataset has been used during the experiments. Experimental results demonstrate that the proposed approach outperforms the previously adopted techniques.

## 1. Introduction

The population of the world is increasing at a rapid pace and resources are limited when considering the growing population. This factor leads humans to develop advanced techniques as part of industrial revolutions to fulfill demands and maintain living standards. These industrial revolutions have changed everything in the world from economies to societies. All the production and consumption patterns of the goods and resources such as fossil fuels and other energy resources have been affected. Smart grids are a part of the modern infrastructure in energy management, which manages the consumption of energy for each consumer or specific area and provide useful information on energy consumption and production [1]. One of the major features of the smart grid is Demand Response Management (DRM). This helps the consumer by providing information about their electricity consumption and methods to improve the energy efficiency that can reduce the cost. Providing information about load forecasting is essential for the consumer as well as for the supplier as it minimizes the gap between supply and demand. Many countries with the help of experts aim to build accurate models to achieve better results in load forecasting [2].

Even though we live in an advanced technology era, the electric power industry still struggles to provide a stable, low-price, and safe power supply to consumers. Fortunately, time series forecasting and prediction algorithms are widely used successfully in real-world applications such as weather forecasting and the financial market. [3]. There are some factors such as weather, holidays, and other unexpected events such as forest fires and earthquakes that can change the electricity consumption patterns. Recently, the COVID-19 pandemic has exposed many flaws in the national structures such as hospitals, energy sectors, and the food supply chain. For example, China is the largest producer of electricity in the world; however, it also lacked the energy supply in some areas of the country during the COVID-19 pandemic just because the system could not predict such kinds of unexpected electricity usage patterns.

Load forecasting is divided into four major categories that are based on the intervals [4]. These include very short-term, short-term, medium-term, and long-term load forecasting. The very short-term load forecasting lasts for a few minutes, the short-term is between 24 h and one week, the medium-term starts from 1 week and can be extended up to few months, and the long-term forecasting is around one year or longer. Generally, short-term and medium-term load forecasting is crucial in the energy sector because the infrastructure is built based on these accurate predictions. There are two major challenges for these forecasting algorithms that are accuracy and prediction stability [5]. In the case of short-term forecasting, accuracy is more important because it deals with day-to-day operations of power delivery, and it is necessary for the system to accurately measure future demands. In the case of medium-term forecasting, the prediction stability is important because the model should be able to predict the precise scheduling of the supplies and maintenance to provide power to the end-users flawlessly [6].

To overcome the problem of accuracy and prediction stability, many statistical and machine learning-based models have been applied in energy forecasting. To achieve better accuracy and prediction stability, it is essential to design a new state-of-the-art forecasting model. Generally, the algorithms that gain the knowledge of current trends are statistical parameter estimation-based models, machine learning-based models using shallow-structured representations, and deep learning-based models. For the statistical methods, correlation and physical information of the infrastructure is required to implement the nonlinear mappings. These models include regression analysis, filtering (e.g., Kalman filter), auto-regression, and moving average. Generally, these methods are derived theoretically and assumptions are made to determine certain parameters helping in mining useful patterns in data to build an empirical method. On the other hand, shallow-structured representation models are more suitable for power forecasting as they operate on self-learning parameters. These models are derived from machine learning methods such as k-Nearest Neighbor (k-NN), Support Vector Machines (SVM), and Decision Trees. Moreover, hybrid methods are used to improve the accuracy and prediction stability of the models [3].

Recently, deep learning-based models have shown promising results in different application domains because of sophisticated state-of-the-art algorithms and network architectures. As the electric industry is shifting towards a new era, deep learning will be playing a more important role in this shift. The deep learning models require high-quality and enormous data to operate and, fortunately, the energy field contains sufficient data that can be used by these models. Some of the deep learning-based models that utilize time-series data to predict power load are Convolutional Neural Networks (CNNs), Recurrent Neural Networks (RNNs), Generative Adversarial Networks (GAN), Graph Neural Networks (GNN), and Deep Belief Networks (DBNs) [7,8].

While different prediction models provide short-term load forecasting in literature, their performance is not at an acceptable level yet. As such, in this study, the main objective is to improve the short-term load forecasting models.

To improve the performance of Recurrent Neural Network-based models, we use the attention mechanism that helps to keep only the most useful information at each stage. The proposed attention mechanism also excels at using two distinct attention layers. First, an input attention layer is utilized in the encoding phase to improve the quality of the outputted context vector. In addition, a temporal attention layer has been used in the decoding phase to effectively decode the context vector and capture longer sequences, thus improving the predictive power of the whole model. This Dual-Attention approach has not been investigated in the energy load forecasting domain, which demonstrates unique characteristics of being volatile, yet is seasonal and follows cyclical patterns.

The contribution of this study is presented as follows:

- A novel deep learning model has been developed for predicting the energy load consumption. The model comprises a dual-stage attention-based deep algorithm where the attention mechanism is applied in both encoder and decoder stages.
- The proposed approach improves the performance of very short-term load forecasting (VSTLF) and short-term load forecasting (STLF) models in terms of assessment metrics, namely Mean Absolute Error (MAE) and Root Mean Squared Error (RMSE).

The paper is organized as follows: Section 2 provides the related work. Section 3 explains the methodology including the LTSM algorithm, Encoder–Decoder Networks, Attention Layer, and Evaluation Metrics. Section 4 presents the experimental results. Section 5 shows the discussion, and Section 6 concludes the paper.

## 2. Related Work

This section discusses relevant studies for forecasting energy consumption based on machine learning, deep learning, and hybrid methods. These studies propose different methodologies for feature extraction and modeling. There are several methods for short-term and medium-term forecasting methods that are discussed in this section.

Previously, machine learning and traditional time series analysis methods have been widely used in the field of smart energy systems. Methods like linear regression and Support Vector Machines have been used. One of the studies [9] used sinusoidal variations as an input for the models like linear regression for the prediction of daily and weekly electricity demands in the Turkish energy market. Another study [10] used Support Vector Machines to predict hourly and daily energy consumption demands. The Random Forest model was used by [11] to predict short-term forecasting. In their study, the author used time-series data to predict the estimated demands and consumption for the energy.

The widespread adoption of Artificial Neural Networks also attracted researchers working in the energy forecasting field. These days, deep learning methods are used in many different application domains because of their nonlinearity and robustness characteristics. The most common methods that are used include Multi-Layer Perceptron (MLP), fuzzy neural network, wavelet neural networks, deep neural networks, and long short-term memory-based methods [6]. Previously mentioned models have accomplished promising results in this field. The reason behind this success over machine learning models is to learn more and high-level features automatically from the data itself. Tian et al. proposed a deep learning model for short-term load forecasting [1]. The model comprises two neural networks, namely Convolutional Neural Networks (CNNs) and Long Short Term Memory Network (LSTM). CNN can capture the local power trend and the similar patterns present in the dataset and the LSTM learns different relationships in multiple timestamps. The CNN that captures the hidden features is integrated with LSTM to improve the accuracy of the model. The proposed model was tested on the real-world dataset and different experiments were performed to validate the proposed model. Experimental results indicate that the model performs better than the others in the literature. Another study [3] proposed a method based on the attention-based encoder–decoder combined with Bayesian optimization to overcome the limitations of the short-term electrical load forecasting such as safety and stability. In this model, the temporal attention layer of the encoder–decoder captures the key features of data and the Bayesian optimization controls the model hyperparameters to ensure the best optimal predictions. The model was tested on real-time load forecasting of American Electric Power and outperformed the previous methods.

To overcome and extract the irregular energy patterns and reduce the translational variance in the power forecasting for residential area consumption, Seok-Jun et al. proposed multi-headed attention and Convolutional Recurrent Neural Network-based deep learning model [12]. To model the transient and impulsive nature of the power demand, it calculates the attention scores using softmax and the dot product in the present network. This model was tested on the University of California Irvine data set that is based on households. The model achieved better performance results. Salah et al. proposed a multi-sequence LSTM-RNN based deep learning model to obtain the optimal configuration of LSTM [6]. According to this study, normal LSTM-based models do not achieve acceptable results in forecasting. To overcome the limitations of LSTM-based models, this study used metaheuristics search-based algorithms that are generally known for being better in search complexity. The results indicate that the proposed model provides better results than the traditional machine learning models.

A short-term prediction forecasting model based on LSTM and Discrete Wavelet Transform was proposed for wind power [13]. To exhibit the behavior of wind, the LSTM based network is designed and, for the decomposition of non-static wind power, the wavelet transform is introduced in the proposed model. The prediction accuracy is better than the previous studies. Moreover, this model provides security and stability for the power network. Another study utilizes LSTM along with the CNN model to predict the energy consumption in residential areas [14]. This model can extract the spatial and temporal features to predict the accurate energy consumption for houses. According to this study, this CNN-LSTM method achieves better prediction for energy consumption than the other previous models. Wang et al. proposed a probabilistic forecasting model for each consumer, and it captures the uncertainty and variability of loads in the near future. In this study, LSTM is used to predict the dependencies in each of the individual profiles and, for validation, pinball loss is calculated instead of MSE [15].

The usefulness of Advanced RNNs based strategies is discussed in another study [16]. It describes multiple strategies for predicting energy consumption and each of them gives insights into unique characteristics of models. The study discusses different level approaches and each level contains a different set of techniques used for energy consumption prediction. Yixing et al. proposed a short-term load forecasting model based on a Gated Recurrent Unit (GRU) neural network. The data of the target users is pre-processed by a clustering algorithm to reduce the noise [17]. The multi-source information is used as an input. To extract the temporal features of the data, GRU neural networks are used. The model calculated absolute percentage error to show the superiority of this model over the others.

Usage of LSTMs in this area has been used extensively. For instance, Abdel-Nasser et al. [18] proposed a solar forecasting model based on LSTMs that overcome the deficiency in previously proposed models in this area. In the previous studies, individual or ensemble forecasting techniques are used. However, this study overcomes these limitations by proposing a solar irradiance forecasting method that is based on LSTM and aggregation function based on Choquet integral. As the LSTM can predict the temporal changes accurately and the Choquet integral can precisely aggregate the inputs via fuzzy measure by modeling the interaction between the inputs [18]. Another study used LSTM-RNN based architecture to precisely forecast the output power of Photovoltaic systems [19]. The above-mentioned proposed studies achieved better results than the previous studies present in the literature. However, the afore-mentioned study has limitations such as the effect of outliers in PV plants and environmental parameters are not discussed in this study. Lin et al. [20] proposed an RNN and LSTM based study to capture the prediction fault in the output power of wind turbines. This architecture aimed to learn the efficient spatio-temporal properties of the output power [20].

Agga et al. [21] proposed two hybrid models CNN-LSTM and ConvLSTM to predict the power consumption of PV plants. These models were compared against the baseline LSTM models for the performance evaluation. Both proposed models were trained on the multivariate datasets fetched from PV plants. The results indicate that the proposed models are much better than the baseline LSTM models [21]. Another study by Wu et al. [22] proposed a hybrid model based on LSTM and a statistical toolkit named Kullback–Leibler divergence (KLD) that accurately evaluates the performance of the turbine and diagnose the faults [22]. This proposed model is tested on the two faulty wind turbines for fault detection and identification purposes. Zhou et al. [23] proposed another hybrid model based on CNN and LSTM to extract the spatial and temporal characteristics of the data [23]. The experiments were made on the UK-DALE open dataset, and the results are much better achieving an accuracy of 98%.

The proposed approach presents a novel solution of Dual-Stage Attention for Recurrent Neural Networks. The approach improves the predictive power of neural networks. In Encoder–Decoder Recurrent Neural Networks, the context vector is a fixed-size temporal representation. The attention approach brings variable-length temporal representations to life. This leads to better predictive power in the case of much longer sequences. The proposed approach uses an input attention mechanism in addition to the temporal attention mechanism to further increase the predictive power of the model. A summary of related studies is provided in Table 1.

## 3. Methodology

This section explains LSTM, encoder–decoder networks, attention concept, and evaluation metrics. Electrical load reading data consist of series of time-stamped electrical load measures as well as some other factors such as voltage and intensity. The electrical load forecasting problem targets an accurate prediction of electrical load in the next time window based on earlier records. In that sense, the problem can be modeled as a regression problem. As the input dimension is high, traditional machine learning approaches are not strong enough to model this problem and more complex models are needed.

### 3.1. LSTM

Recurrent Neural Networks-based deep models show promising outcomes in the case of time-series related problems such as natural language processing (NLP), musical information retrieval (MIR), and speech recognition (SR) [24]. The architecture offers to keep track of previous information by using internal memory and recurrent connections of neurons [25]. RNNs do not perform well in the case of capturing long-term structures. The information must survive across many neurons until reaching the current processing neuron. However, in the back-propagation phase, this long-term information slowly converges to zero and is finally lost because the value gets multiplied by such small values many times. This problem is called the vanishing gradients problem. Newer methods such as LSTM and GRU networks offer an improved design and better performance on representing longer sequences and dealing with the vanishing gradients problem.

**Figure 1:**
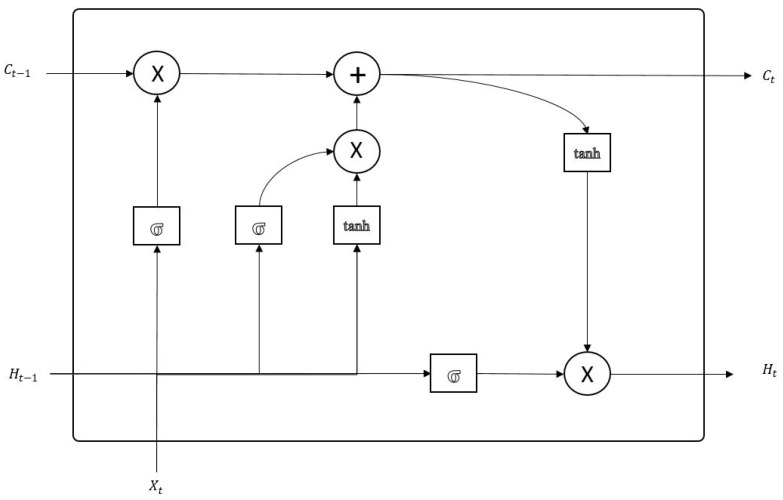
Figure LSTM.

Long-Short Term Memory (LSTM) networks improve the standard RNN structure through combining short-term memory with a long-term memory concept and overcome the vanishing gradients problem [26]. This long-term memory concept makes important information pass neurons without modification. Unlike an RNN cell, LSTM cells decide whether the past information should be passed using a three-gate structure as shown in Figure 1.

The forget gate is used to decide whether the previous cell state should be preserved or not. The gate uses a tanh function to regulate the combined vector of the previous cell’s hidden state $h_{t}-1$ and the current cell’s input $X_{t}$. Outputs close to 0 lead to previous cell states being forgotten. The definition includes $W_{f}$ weight vector and $b_{f}$ bias vector and is given in Equation (1):

(1) $$f_{t}=\sigma \left(W_{f}*\left[h_{t}-1,X_{t}\right]+b_{f}\right)$$

Th input gate decides on the current cell state with respect to previous cell hidden state $h_{t}-1$ and current cell input $X_{t}$. The gate combines a tanh output of both inputs with a sigmoid output and produces the current cell state $C_{t}$, as shown in Equation (2):

(2) $$i_{t}=\sigma \left(W_{i}*\left[h_{t-1},X_{t}\right]+b_{i}\right)$$

Finally, the output gate decides on the hidden state of the current cell $H_{t}$ with respect to current cell state Ct from the input gate and the sigmoid output of current cell input $X_{t}$. The output gate $O_{t}$, the new cell state $C_{t}$, and the new hidden state $h_{t}$ are calculated based on Equations (2)–(4):

(3) $$C_{t}=f_{t}*C_{t}-1+i_{t}*\left(tanh\left(W_{c}*\left[h_{t-1},X_{t}\right]+b_{c}\right)\right)$$

(4) $$o_{t}=\sigma \left(W_{o}*\left[h_{t-1},X_{t}\right]+b_{o}\right)$$

(5) $$h_{t}=\sigma_{t}*tanh\left(C_{t}\right)$$

The hidden state $h_{t}$ and the new cell state $C_{t}$ are passed to the next cell. In addition, the hidden state $h_{t}$ is used for prediction.

The GRU architecture is proposed in 2014 by Cho et al. [27] and offers the use of two gates inside a cell. While it is reported to represent longer sequences better than standard RNN implementation, it offers a similar performance against LSTM architectures [28].

### 3.2. Encoder–Decoder Networks

**Figure 2:**
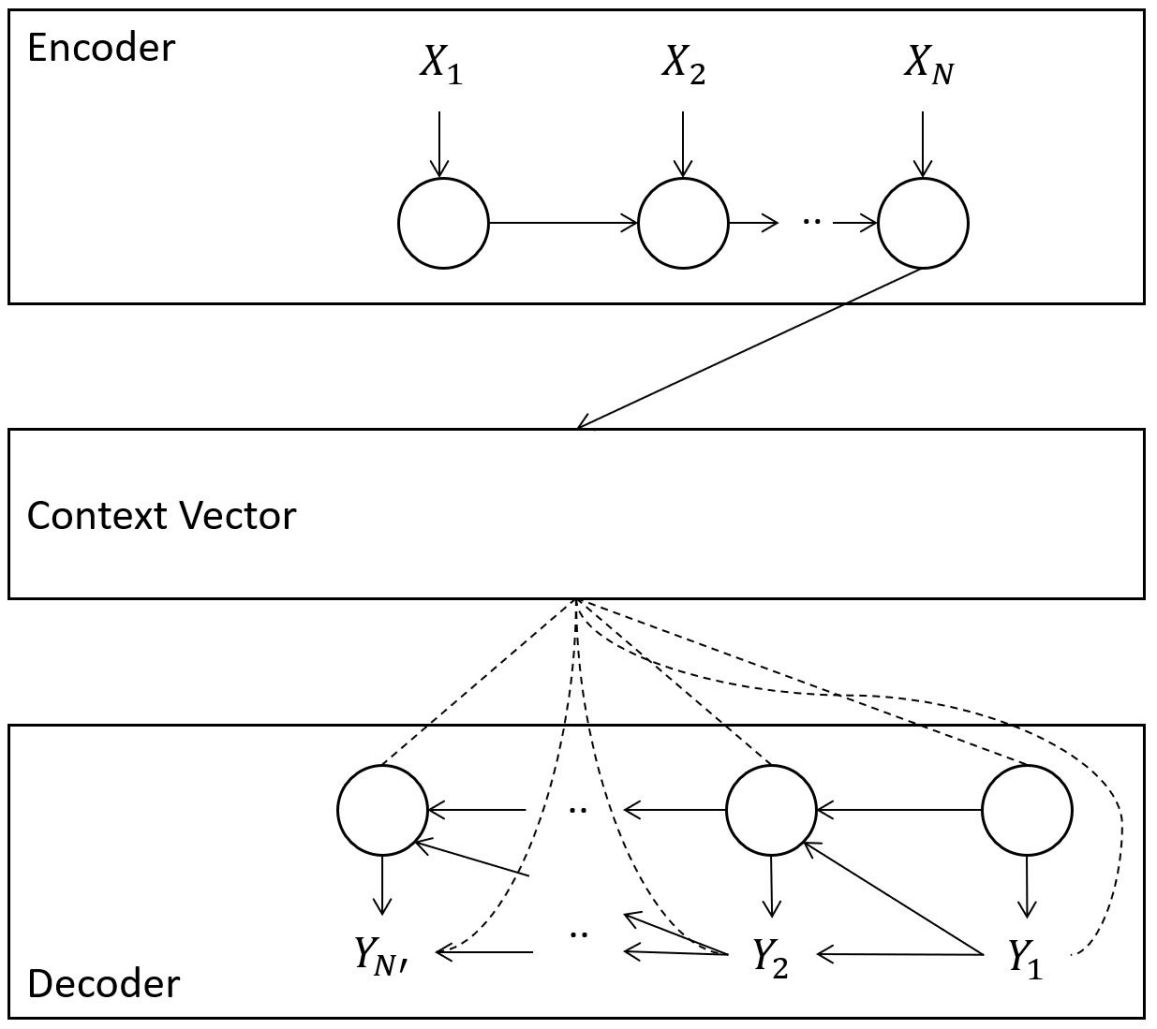
Encoder–decoder model architecture.

Both LSTM and RNN networks are sensitive to input length and expect a standard length of an input variable. In sequence-to-sequence problems such as multi-step time series forecasting, this brings a new challenge. Encoder–decoder networks, on the other hand, can accept different size input variables [3]. As shown in Figure 2, a standard encoder–decoder cell consists of two models called encoder and decoder. The encoding phase consists of encoding of a variable size input vector *X* and produce a fixed size context vector *C*. The context vector is a powerful and compact representation of data features. Given a time series vector $X=(x_{1},x_{2},\ldots ,x_{t})$, where $x_{t}$ ϵ $R^{n}$, mapping from *X* to context vector *C* can be conducted with an activation function $f_{1}$ such as RNN, LSTM, or GRU. In the decoder phase, the compact feature representation is decoded. The inputs are the last hidden state $h_{t-1}$ from the encoder phase and the context vector *C*. The output of the decoding phase is a prediction vector or, in other words, the output vector.

### 3.3. Attention Layer

The encoder–decoder approach has a limitation in the case of long sequences. The model performance starts to deteriorate when longer sequences arrive. The attention approach increases weights for more relevant information [29]. From a technical point of view, attention makes it possible for the context vector to create shortcuts between hidden states and the input vector. Thus, instead of creating an entire context vector out of the last encoder hidden state, a context vector that can remember much longer sequences can be produced.

Qin et al. proposed a method based on a dual-stage attention-based recurrent neural network to overcome the problems present in nonlinear autoregressive exogenous (NARX) [5]. The NARX methods have been present in the literature for a long time, but most of the models cannot properly capture long-term temporal dependencies, and it is also difficult for such models to select a relevant series for the prediction purpose. As such, to resolve these issues, they proposed a model in which the first stage selects the relevant series for input data by using an attention mechanism. In the second step, the encoder hidden states are selected by using a temporal attention mechanism. Their model was tested on the datasets of SML 2010 and NASDAQ 100 Stock and outperformed the other models.

**Figure 3:**
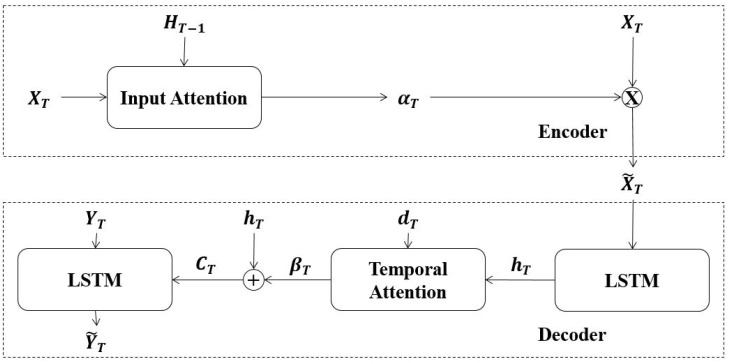
Proposed dual-stage attention architecture.

In this study, we investigate the applicability of the dual-stage attention approach to overcome the context vector limitations and apply this algorithm to improve the predictive power of short-term electrical load forecasting models. The architectural view of the attention approach is presented in Figure 3.

The first stage includes the application of an attention layer on direct input series to select weighted feature vector $\tilde{X_{t}}$. The input series of $X_{t}$ are fed into an input attention layer. Attention weights of $\alpha_{T}$ are obtained with respect to previous hidden state $H_{t}$ in the encoder phase, as seen in Equations (6) and (7):

(6) $$e_{T}=V_{e}^{T}tanh\left(W_{e}\left[h_{t-1};s_{t-1}\right]+U_{e}X\right)$$

(7) $$\alpha_{T}=\frac{exp(e_{T})}{\sum exp(e_{T})}$$

Later, attention weights are multiplied with the input vector to obtain the encoded input (context) vector. To overcome the fixed-width representation limitation of the context vector, the decoder phase is also accompanied by an attention mechanism. The context vector is directly supplied to an LSTM unit present in the decoding phase. LSTM unit’s hidden state $h_{T}$ and cell state are fed into the temporal attention layer. The final attention weights $\beta_{T}$ are summed again with hidden state $h_{T}$ to form decoder context vector $C_{T}$. Finally, decoder context vector and $Y_{t}$ are supplied as an input to the decoder LSTM to make the next prediction $\tilde{Y_{t}}$.

### 3.4. Evaluation Metrics

To assess the predictive performance of the proposed model, three metrics, namely Mean Absolute Error (MAE) and the Root Mean Square Error (RMSE), were applied. The MAE measurement is the average absolute difference between predicted and actual values. The RMSE is the square root of the MAE. The error measures are defined in Equations (8) and (9). *N* is the length of the sample set, $y_{p}$ stands for the predicted value, and $y_{c}$ stands for the actual value:

(8) $$MAE=\left(1/N\right)*\sum_{L=1}^{N}\left|\left(y_{p}-y_{c}\right)\right|$$

(9) $$RMSE=\sqrt{\left(1/N\right)*\sum_{L=1}^{N}|{\left(y_{p}-y_{c}\right)}^{2}}|$$

## 4. Experimental Results

### 4.1. Dataset

We have used the UCI household electric power consumption (HEPC) dataset (https://archive.ics.uci.edu/ml/datasets/individual+household+electric+power+consumption, accessed on 10 August 2021) to assess the predictive performance of the proposed model. HEPC is a minutely power consumption dataset recorded between 2006 and 2010 for a single house. As shown in Table 2, the HEPC dataset has seven attributes, namely global active power (power lost due to use in devices), global reactive power (power lost due to transfer on cables), voltage, global intensity, and three sub-meterings for kitchen, laundry, and climate control unit. The hourly energy demand generation (HEDG) dataset (https://www.kaggle.com/nicholasjhana/energy-consumption-generation-prices-and-weather, accessed on 10 August 2021) is also used to demonstrate the general applicability of the proposed model. The HEDG dataset offers four years of electrical consumption, generation, pricing, and weather data for Spain in hourly precision, suitable for STLF analysis.

### 4.2. Experimental Environment

The proposed deep models are implemented in Python using Keras framework with version 2.2.4. The model is trained on Nvidia 3090 GPU with CUDA version 8. Eight GPUs were used in parallel to accelerate the overall training process. The graphics were obtained using plotting libraries matplotlib and seaborn.

#### Data Preparation

The values present in the HEPC dataset demonstrate different scales amongst different features [30]. This leads to one feature being dominant and data analysis being inefficient. The success of machine learning analysis depends on the quality of data and representation. In terms of electrical load forecasting analysis, the proposed data features employ equal significance. On time series based analyses, min-max normalization is reported to show better performance with respect to its counterparts [31,32]. Thus, to normalize the features, we decided to employ min-max normalization. The normalization is calculated based on Equation (10):

(10) $$\tilde{X}=\frac{x-min}{max-min}$$

X shows the input variable, min and max values point to the lowest and highest points present in series, and $\tilde{X}$ indicates the normalized value. The dataset also contained some missing values. The percentage of missing values is only smaller than 0.0001%. Thus, the timesteps with missing values are kept, but they are filled with the previous days’ recordings. The minute representation of the dataset is also merged to create an hourly time series data. To comply with the STLF analysis, we did created hourly and daily versions of the original minute time series dataset.

### 4.3. Hyperparameter Optimization

A correct hyperparameter combination is crucial to obtain an efficient deep neural network. The hyperparameters for the proposed Dual-Stage Attention network for short-term electrical load forecasting have been selected using heuristic optimization methods and the resulting combinations are depicted in Table 3. The parameters are searched between a pre-determined search space and only one parameter is adjusted at a time. Starting with optimizers, the popular Adam optimizer provided the best results. Next, the learning rate has been decided where values between 0.001 and 0.1 are evaluated and 0.001 is decided to have the best performance. The neural size of both encoder and decoder stages are evaluated between the range of [16–512] and our experiments point to an optimal neural size of 128. Batch size and the number of epochs are decided with trial and error with respect to hardware limitations. Thus, heuristic experiments are run through 200 epochs with a batch size of 64.

### 4.4. Model Performance Based on 10-Fold Cross Validation Approach

The predictive performance of the proposed model has been assessed for both VSTLF, which is the prediction for few minutes, and also, for STLF, which stands for prediction of time frames ranging from an hour to a week. Experiments are run using 10-fold cross validation approach. For the statistical significance analysis, we have adopted the Wilcoxon test as the Wilcoxon test offers better evidence than its counterparts such as a *t*-test. Table 4 presents the error rates of the proposed study for different time windows concerning the RMSE and MAE metrics. A Dual-Stage attention-based deep model demonstrates remarkable predictive accuracy for the scales of both VSTLF and STLF. The results are also competitive in case of longer time windows such as MTLF (Medium-term load forecasting) and LTLF (Long-term load forecasting).

**Figure 4:**
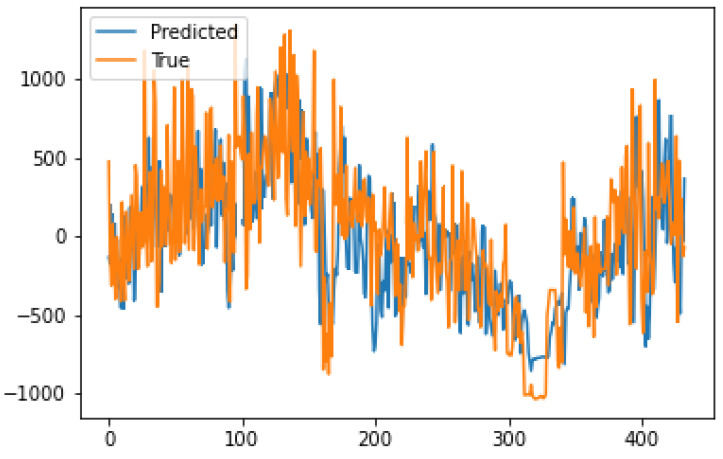
Hourly forecasting performance.

The forecasting performance of the model for hourly analysis is visualized in Figure 4. The model shows its power in detecting the change in momentum as well as offering high performance even at noisy scales of SLTF.

To demonstrate the performance of the proposed architecture, baseline LSTM and GRU models are implemented and their respective MAE and RMSE scores are obtained. Table 5 compares STLF forecasting performance of the proposed architecture with baseline LSTM and GRU architectures. The table is also expanded with the SLTF forecasting scores of the newest studies in the field. The proposed dual-stage attention algorithm shows a clear improvement in STLF forecasting performance against both baseline implementations and previous studies.

To prove that the proposed model can be generalized to other datasets, we also trained the model with the HEDG dataset, and the results are given in Table 6. Baseline GRU produces comparable results against baseline LSTM, but both models are outperformed by the proposed Dual Attention architecture.

## 5. Discussion

To the best of our knowledge, this is the first study that applied a dual-stage attention-based RNN model for residential energy consumption in the STLF time span. The performance of the proposed model can be improved by using other deep learning-based algorithms and networks. Since this is an emerging research field, new algorithms and models are currently being developed by deep learning researchers. For instance, transformer algorithms such as BERT and GPT-3 can be applied to predict the energy consumption with higher accuracy. In addition to the LSTM-based models, we have also performed experiments with the GRU-based prediction model. Compared to the LSTM algorithm, GRU is relatively less complex and, therefore, it mostly trains faster than the LSTM-based models. While GRU has two gates (i.e., reset and update), LSTM has mainly three gates (i.e., input, output, and forget gates). As such, GRUs can provide comparable performance on smaller training datasets and their structures can be easily modified if additional inputs are needed. During our experiments, we observed that our proposed model provides better performance than the GRU-based prediction model. Since the proposed model is more complex, maintainability and reusability aspects are limited when compared to the GRU-based prediction models. Although we have used different network types of LSTM algorithm and compared the performance with other LSTM-based models, there might be more LSTM-based network types that we have not investigated in this study. Therefore, researchers might consider applying the other LSTM-based network types that have not been evaluated in this study.

The 10-fold cross-validation approach, which is a widely-used evaluation strategy, has been used during the experiments to evaluate the performance of the models. However, different evaluation approaches might also be preferred, and we do not expect much change in the performance of models. Another issue is that the performance of the proposed model can be different on other datasets; therefore, we expect some minor performance change when the proposed model is applied on new energy consumption datasets. To show the applicability and generalizability of the proposed model, we have also performed experiments on a second dataset. However, it would be more beneficial to execute all the experiments on larger and more publicly available datasets. Researchers or practitioners can apply the proposed model on their own datasets and evaluate the performance easily. It is also possible to adjust some parameters for fine-tuning the model for the underlying prediction problem.

We derived all conclusions based on the experimental results shown in tables and figures and, therefore, avoided the subjective interpretation of the results of researchers in this study. In addition, we aimed to minimize the researcher bias on the selection of algorithms in this research. Therefore, authors carefully considered all the available algorithms and models in literature and selected them after discussing the implications of them in different meetings. If some models have not been documented in scientific literature and have been applied only in industry, those models have not been considered in this research. However, since the datasets are publicly available, researchers and practitioners can easily perform new experiments to demonstrate the superiority of their models.

The applied pre-processing techniques, dataset characteristics, and mechanisms that prevent overfitting and training configuration might have affected the performance of our prediction models. Therefore, future research might consider applying different feature engineering techniques, different regularization/randomization steps, newly developed deep learning models, and additional optimization approaches to improve the overall performance.

Current research did not aim to build models that are explainable, as such, researchers might also consider developing new interpretable machine learning models for this problem. Since the explainability of the deep learning-based models is limited, a new research on this issue can be a challenging research direction. To address this challenge, it is advised to discuss with the domain experts how they would like to be informed with new explainable prediction models. For instance, while explainability by visualization might be an option for a domain expert, others might prefer explainability by examples. There are many different approaches for explainability and, therefore, this should be discussed with the end-users.

## 6. Conclusions

Accurate estimation of residential energy consumption has several benefits for energy service providers. There are many factors that affect the performance of these prediction models. This study aimed to improve the performance of very short-term load forecasting and short-term load forecasting models using deep learning algorithms. This study presented a novel load forecasting approach that utilizes input and temporal attention in order to overcome context vector limitations and achieve higher short-term load forecasting performance due to lower error values when compared to other studies. Particularly, the model based on the dual-stage attention-based RNN algorithm has been built and evaluated on a public dataset.

It was shown that the proposed novel model provides better performance than the other competing algorithms reported in literature. Less complex prediction models can also be built using the GRU algorithm; however, the performance is relatively lower in such a case. If modifiability and maintainability of the models are more important than the performance of the models, simpler models could be an option.

The Dual-Stage Attention-based LSTM model provided superior performance compared to the other prediction models. The models that we implemented using Keras framework are reusable, and new experiments can be easily performed when new prediction datasets are available. There were also other options for implementation of the proposed models; however, since Keras provided simpler APIs, we designed and implemented the overall system based on Keras framework. We observed that the development time was also very effective and efficient thanks to the well-designed APIs of the Keras platform.

Future work will focus on the development of Explainable Artificial Intelligence (XAI) models for the underlying problem. In addition, different network architectures based on deep learning will be investigated to improve the overall performance reported in this research. Both model-specific and model-agnostic approaches will be evaluated, and the best strategy will be determined. We also aim to work closely with domain experts because the explainability aspect should be considered from the domain expert perspective as well.

## Figures and Tables

**Table 1:** Related work summary.

	Dataset	Method	Evaluation Metric
Tian et al. (2018)	Entsoe: Italy	LSTM CNN	MAE, MAPE, RMSE
Jin et al. (2021)	AEP	Temporal Att. LSTM	MAE, RMSE, MAPE
Bouktif et al. (2018)	RTE	LSTM RNN	MAE, RMSE
Bouktif et al. (2020)	RTE	LSTM	MAE, RMSE
Proposed Approach	UCI HEPC	Dual Att. LSTM	MAE, RMSE

**Table 2:** UCI household dataset feature statistics.

Attribute	Definition	Average	Std. Dev.
Global active power (kW)	Power consumed on real devices	1.0916	1.0573
Global reactive power (kW)	Power consumed on transmission	0.1237	0.1127
Voltage (V)	Voltage meterings	240.8399	3.2400
Global Intensity (A)	Intensity meterings	4.6278	4.4444
Kitchen (Wh)	Sub-metering	1.1219	6.1530
Laundry (Wh)	Sub-metering	1.2985	5.8220
Climate Control (Wh)	Sub-metering	6.4584	8.4372

**Table 3:** Hyperparameter search space and best hyperparameters.

Hyperparameter	Search Space	Selected Value
Learning Rate	[0.01–0.001]	0.001
Epoch	[10–400]	200
Optimizer	[Adam, Adagrad, Rmsprop]	Adam
Encoder Neurons	[16–512]	128
Decoder Neurons	[16–512]	128

**Table 4:** The experimental results for different time windows.

Time Window	1 min	15 min	1 h	1 Day	1 Week
Type	VSTLF	VSTLF	STLF	MTLF	LTLF
MAE	260.14	290.82	240.14	298.65	332.10
RMSE	328.20	320.24	300.18	360.86	398.26

**Table 5:** Performance comparison with previous studies.

Model	Type	MAE	RMSE
Dual Attention LSTM (Proposed Model)	STLF	240.14	300.18
LSTM	STLF	652.2	810.12
GRU	STLF	712.24	880.16
LSTM_GA [33]	STLF	231.50	311.44
GA_LSTM [6]	STLF	249.53	341.40
Temporal Attention [3]	STLF	458.93	550.39
CNN-LSTM [1]	STLF	692.14	1134.17

**Table 6:** STLF Performance in the HEDG dataset.

Model	Type	MAE	RMSE
Dual Attention LSTM (Proposed Model)	STLF	314.08	316.22
LSTM	STLF	750.20	824.46
GRU	STLF	748.74	818.86

## Data Availability

UCI Household Electric Power Consumption dataset is available at: https://archive.ics.uci.edu/ml/datasets/individual+household+electric+power+consumption (accessed on 22 August 2021).
